# Investigation on eggshell apex abnormality (EAA) syndrome in France: isolation of *Mycoplasma synoviae* is frequently associated with *Mycoplasma pullorum*

**DOI:** 10.1186/s12917-020-02487-0

**Published:** 2020-08-05

**Authors:** M. Cisneros-Tamayo, I. Kempf, J. Coton, V. Michel, S. Bougeard, C. de Boisséson, P. Lucas, M.-H. Bäyon-Auboyer, G. Chiron, C. Mindus, A. V. Gautier-Bouchardon

**Affiliations:** 1 Mycoplasmology, Bacteriology and Antimicrobial Resistance Unit, Ploufragan-Plouzané-Niort Laboratory, French Agency for Food, Environmental and Occupational Health and Safety (ANSES), Ploufragan, France; 2 Facultad de Medicina Veterinaria y Zootecnia, Universidad Central del Ecuador, Quito, Ecuador; 3 Epidemiology and Welfare in Poultry and Rabbits, Ploufragan-Plouzané-Niort Laboratory, ANSES, Ploufragan, France; 4 Animal Welfare National Coordination, ANSES, Niort, France; 5 Epidemiology and Welfare in Pigs, Ploufragan-Plouzané-Niort Laboratory, ANSES, Ploufragan, France; 6 Viral Genetics and Biosafety unit, Ploufragan-Plouzané-Niort Laboratory, ANSES, Ploufragan, France; 7 Labocea, Ploufragan, France; 8 ITAVI, Lyon, France

**Keywords:** *Mycoplasma synoviae*, Eggshell apex abnormalities, Layers, MALDI-TOF, *Mycoplasma pullorum*

## Abstract

**Background:**

*Mycoplasma synoviae* (MS) is known to cause Eggshell Apex Abnormality (EAA) syndrome characterized by an altered shell surface with increased translucency on the apex. However, no large-scale studies have been conducted to obtain prevalence data of EAA and MS isolates associated to this syndrome. This manuscript reports the results of two field studies performed in the French poultry industry (2015–2017): focusing mainly on investigation of presence and prevalence of EAA in different types of laying hen flocks (phase 1), and isolation of MS strains from EAA-infected flocks (phase 2).

**Results:**

The first survey included 77 farms of commercial layers in three French egg-production regions, hosting 40 flocks in alternative systems (ALT) and 56 in furnished cages (FC). Seven flocks (4 FC and 3 ALT) presented EAA clinical signs, giving a prevalence of 7.3% in this studied sample. A second independent field study was conducted to identify MS by in vitro cultivation and PCR in samples from 28 flocks with clinical signs of EAA. Different types of biological specimens were collected in EAA-affected flocks and submitted to the laboratory. *M. synoviae* was detected in 25/28 flocks, from both production systems (5/5 ALT and 20/23 FC). Detection of MS was significantly higher in tracheal swabs (59%) than in cloacal (10.5%), albumen (3.6%) and egg yolk (1.1%) swabs. It is worth to mention that attempts to clone MS from positive samples were often hampered by the presence of another *Mycoplasma* species, which showed fast growing behaviour in the selective media used in this study (Frey Medium 4 and Frey Medium 4 supplemented with erythromycin). The use of MALDI-TOF mass spectrometry in combination with next-generation sequencing (NGS) results allowed the identification of this fast growing mycoplasma as *Mycoplasma pullorum*, which was detected in 14 of the 25 (56%) MS-positive flocks.

**Conclusions:**

These results confirmed the presence of the EAA syndrome in MS-positive flocks of layers in France, reared in different regions and in different production systems (ALT and FC). Studies need to be conducted to test whether *M. pullorum* may influence the expression of clinical signs of EAA in MS-infected layer farms.

## Background

Infectious synovitis was first described and associated with mycoplasma infection in the USA during the early 1950’s [1] and the causative organism was designated later as *Mycoplasma synoviae* [2]. It is a cosmopolitan microorganism in poultry production. The clinical signs of MS infection can be different according to its tropism and poultry categories. *M. synoviae* infection most frequently occurs as a subclinical upper respiratory infection, but more severe clinical signs and lesions may be observed when MS is associated with *Escherichia coli* [3], Newcastle disease or infectious bronchitis [4–6], or viruses that may cause immune suppression such as bursal disease virus [7] in chicken. *M. synoviae* can also induce infectious synovitis in chickens and turkeys [1, 8]. In addition to the acute respiratory and/or articular lesions, MS infections often result in reduced growth, production, and hatchability [9, 10]. Feberwee and collaborators [5] described the association between the presence of MS in the oviduct and the production of eggs with eggshell apex abnormalities (EAA) in layers, characterized by an altered shell surface, shell thinning, increased translucency (detectable macroscopically, particularly at candling), and the occurrence of cracks and breaks. Eggshell lesions are confined to a region of approximately 2 cm from the apex (top cone of the egg). This egg alteration is exacerbated by the association of MS and infectious bronchitis virus [5, 11]. The EAA syndrome has been described in several countries [5, 10, 12, 13] and was first reported in France in 2009 [14]. The diagnosis of EAA syndrome due to MS infection in layers is initially based on epidemiological information at the farm level. Direct diagnostic confirmation can be achieved by bacteriological isolation and/or molecular assays such as MS-specific polymerase chain reaction (PCR) tests [8, 15]. Then, several serological tests can be applied for indirect diagnosis of MS infection and according to the World Organisation for Animal Health (OIE), the rapid serum agglutination (RSA) and enzyme-linked immunosorbent assay (ELISA) tests are the most commonly serological tests used for diagnosis [16].

The wide range of MS clinical signs and the multiple exacerbating factors such as other respiratory agents and stress challenges, induce a high economic impact in the poultry industry [15, 17]. Endemic infection in commercial layer farms persists because of vertical and horizontal transmission of MS. Once contaminated, birds may carry MS for the rest of their life [18]. Mycoplasmas lack cell wall, and indirect transmission is rather unexpected for wall-less bacteria, which are supposed to be sensitive to osmotic shock, heating or chemical treatment. Biofilm formation has been evidenced in several *Mycoplasma* species [19, 20] including *M. gallisepticum* [21], but not reported so far in MS. Biofilms may be involved in persistence of mycoplasmas (by increasing resistance to antimicrobials, immune responses, heat and desiccation) and in establishment of chronic infections. *M. synoviae* may persist on feathers for up to two or 3 days at room temperature and ten to 21 days under dry conditions at 20 °C [22]. The presence of MS in poultry farms is frequent despite the control measures and biosafety regulations implemented in different countries, mainly in grandparents stocks and breeders. An official control for MS has been implemented in The Netherlands where the stamping out is mandatory for MS positive breeder flocks [23]. In other countries, biosecurity measures, monitoring and diagnosis, antibiotic therapy, vaccination with commercial or autogenous vaccines are considered as control tools [15, 23–25]. Several studies reported temporary effect of antimicrobial treatments in EAA-affected layer flocks, with a decreased number of broken or downgraded eggs during treatment, but with a disappearance of this effect one to 2 weeks after the end of treatment [5, 12, 26, 27].

There is limited literature on the global prevalence of MS in layers. Some studies reported that MS is found in 73% of layer flocks in the Netherlands [28], 90% in China [29], 40.3% in Portugal [10], 72.7% in Brazil [11], 69% in Australia [16] and 68% in France [18]. However, to our knowledge, no prevalence data for the EAA syndrome (production of eggs with eggshell apex abnormalities in laying-hen flocks) is available.

France is the leading table egg producer in Europe with 46 millions of commercial layers housed in 2100 farms and the main production is brown eggs (60%) [30]. Laying farms are mainly located in Bretagne (42%) and Pays de la Loire (11%) (Northwest of France), Nord-Pas-de-Calais and Picardie (11%) (North of France) and Rhône-Alpes (9%) (Southeast of France). French eggs are produced in furnished cages (69%) and alternative systems (barn, free range and organic production system, 31%) [30]. The aim of this study was to evaluate the status of the EAA syndrome among brown-egg layer farms in France, in the main egg-producing regions (Bretagne, Pays de la Loire and Rhône-Alpes), in flocks housed in furnished cages or alternative systems. In the first phase, the prevalence of this syndrome was calculated during a 12-month field survey (2015–2016). In the second phase, an independent 30-month laboratory study was performed, collecting samples from farms with EAA clinical signs for MS isolation and identification (2015–2017).

## Results

### Field survey results (phase 1): identification of farms with the EAA syndrome

Among the 126 farms selected and contacted for the study, 49 did not want to fill the questionnaire about the EAA syndrome and were not included further in the study. The reduction in the number of farms participating in the survey did not significantly affect the proportions of farms in the different production systems and regions. Thus from May 2015 to May 2016, the 77 remaining layer farms were visited, and filled the questionnaire about the EAA syndrome, for a total of 96 flocks. According to the survey results, 16 out of 77 farmers questioned (farm prevalence: 20.7%, Confidence Interval (CI) = 12.6–31.8) had observed the EAA syndrome in at least one flock under production during the last 5 years (former flocks) (Table 1); on the contrary seven flocks presented EAA clinical signs at the time of visit (current flocks), giving an EAA-positive flock prevalence of 7.3% (CI = 3.2–14.9) in this studied sample. Altogether, EAA was reported in 22 of the 77 farms (farm prevalence: 28.6%, CI = 9.1–40.1) in former or current flocks, and one farm, located in Auvergne-Rhône-Alpes, experienced recurrent cases (Table 1).

**Table 1:** Distribution of EAA cases in the 22 affected farms (96 flocks) according to the region and the production system

Region	No. of visited				EAA cases in					
	Farms	Flocks	Flocks in ALT^**a**^	Flocks in FC^**a**^	Current flocks			Former flocks		
					ALT^**a**^	FC^**a**^	Total	ALT^**a**^	FC^**a**^	Total
**Bretagne**	**52**	**69**	**24**	**45**	**0**	**4**	**4**	**3**	**8**	**11**
**Pays de la Loire**	**8**	**9**	**6**	**3**	**0**	**0**	**0**	**0**	**0**	**0**
**Auvergne-Rhône-Alpes**	**17**	**18**	**10**	**8**	**3**^**b**^	**0**	**3**	**1**^**b**^	**4**	**5**
**TOTAL**	**77**	**96**	**40**	**56**	**3**	**4**	**7**	**4**	**12**	**16**

^a^*FC* furnished cages, *ALT* alternative systems (barn, free-range, organic)

^b^ one farm with one current and one former EAA-positive flock

### Presence of EAA syndrome among different production systems and regions

In total, 40 flocks in alternative systems (ALT) and 56 flocks in furnished cages (FC) were included in the survey. These farms were located in three regions: Bretagne (24 ALT and 45 FC), Pays de la Loire (6 ALT and 3 FC) and Auvergne-Rhône-Alpes (10 ALT and 8 FC). In Bretagne, four flocks in FC out of the 69 visited flocks were EAA-positive (flock prevalence: 5.8%, CI = 1.8–14.9). No EAA-positive flock in ALT or FC production systems was reported in Pays de la Loire. Auvergne-Rhône-Alpes presented the highest prevalence, with three EAA-positive flocks in ALT production systems out of the 18 visited flocks (flock prevalence: 16.6%, CI = 4.4–42.2) (Table 1).

Results of EAA prevalence in former flocks (last 5 years) were different between both production systems. In Bretagne 11/52 poultry farmers (farm prevalence: 21.1%, CI = 11.5–35.0) confirmed the occurence of the EAA syndrome in former flocks: eight farms in FC and three farms in ALT systems. In Auvergne-Rhône-Alpes, 5/17 farmers (farm prevalence: 29%, CI = 11.3–55.9) reported EAA clinical signs in some of their former flocks (4 FC and 1 ALT). None of the eight farms visited in Pays de la Loire reported EAA clinical signs in former flocks (Table 1).

### Age for onset of EAA clinical signs

Among the 22 farms with current or former EAA-positive flocks, 16 farms provided information about age of hens at the start of EAA clinical signs. Abnormal eggs appeared before the production peak (93–95% of production) in two farms with FC (farm prevalence: 12.5%), between 24 and 35 weeks of age in eight farms (farm prevalence: 50%; 4 ALT and 4 FC), and between 40 and 60 weeks of age in six farms (farm prevalence: 37.5%; 3 ALT and 3 FC).

### Vaccination use as control tool for the EAA syndrome

Among the farms with at least one former or current experience of EAA, 16 farmers answered about vaccination practice**.** According to farmers, none of the current EAA-positive flocks were MS-vaccinated. Twelve farmers did not use vaccines to control MS and four EAA-positive farms had used vaccines as a control tool in former positive flocks: autogenous vaccines prepared with an inactivated MS isolate (two farms) and a commercial live vaccine (two farms).

### Use of laboratory analyses for MS monitoring

Survey results showed that diagnosis and/or control of mycoplasmosis via laboratory investigation was not a common practice for the farmers surveyed. The monitoring and tracking with serological and/or molecular tests, as suggested by the OIE (2008), was applied in only 11 EAA-positive farms out of the 77 visited farms (14.2%). In details, three farmers did not provide information about the test used for MS infection detection, two farms were analyzed by PCR methodology (one MS-positive farm), six farms used serology (three MS-positive and one MS-negative farms; results not reported for two farms).

### Statistical analysis of the variables studied

**Fig. 1:**
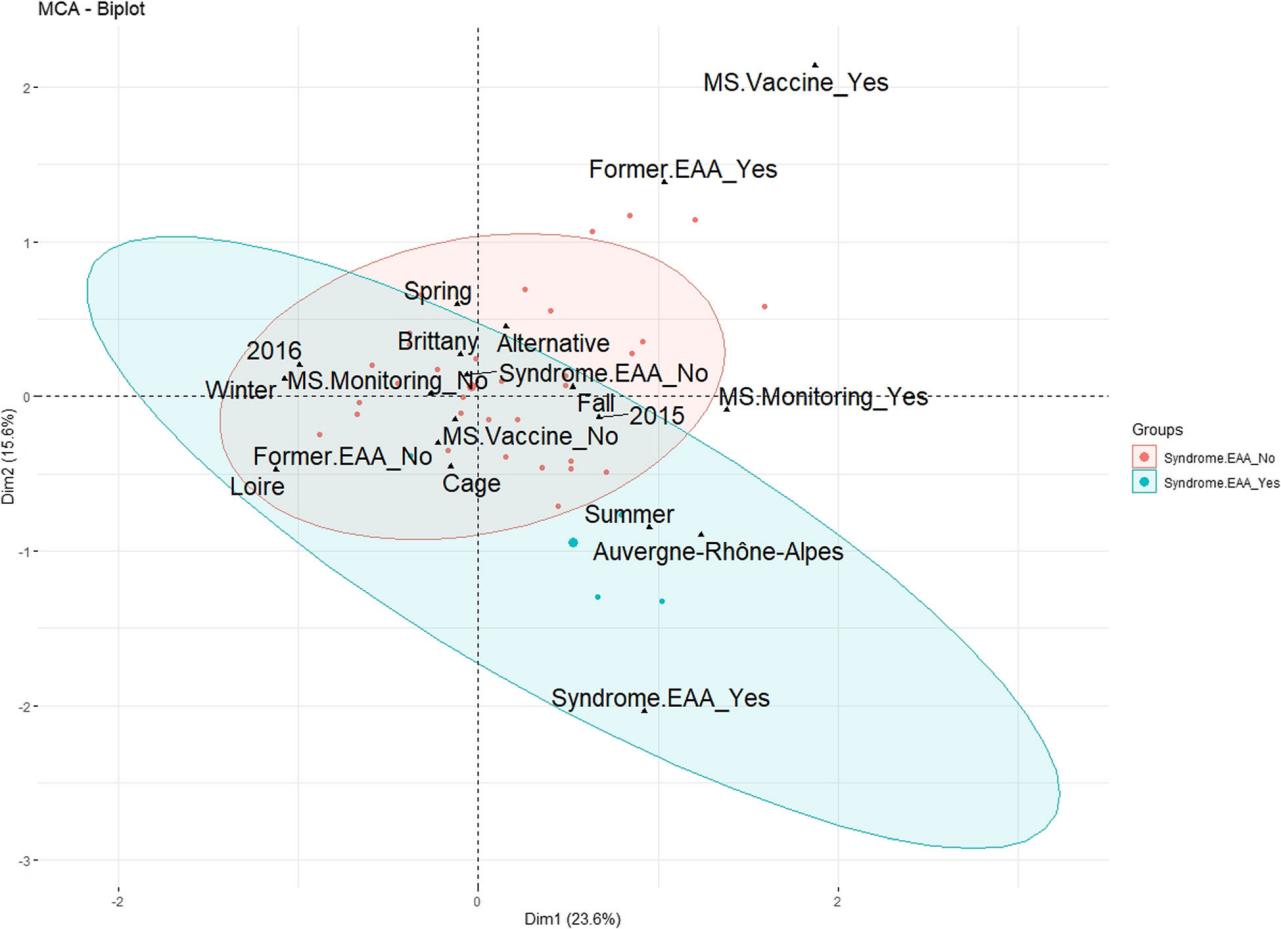
Biplot of the Multiple Correspondence Analysis (MCA). Observations are represented as dots whose color depends on the EAA status (no EEA syndrome in red, EAA syndrome in blue); 95% ellipses are plotted around the observations that belong to each of the two categories of the variable “EAA.syndrome” (i.e., “yes”, “no”). Variables: Type of farm (Alternative or Cage), Area (Brittany, Loire, Auvergne-Rhône-Alpes), Season (Spring, Summer, Fall, Winter), Year (2015, 2016), Former.EAA (Yes, No), Syndrome.EAA (Yes, No), MS.Vaccine (Yes, No), MS.Monitoring (Yes, No)

**Fig. 2:**
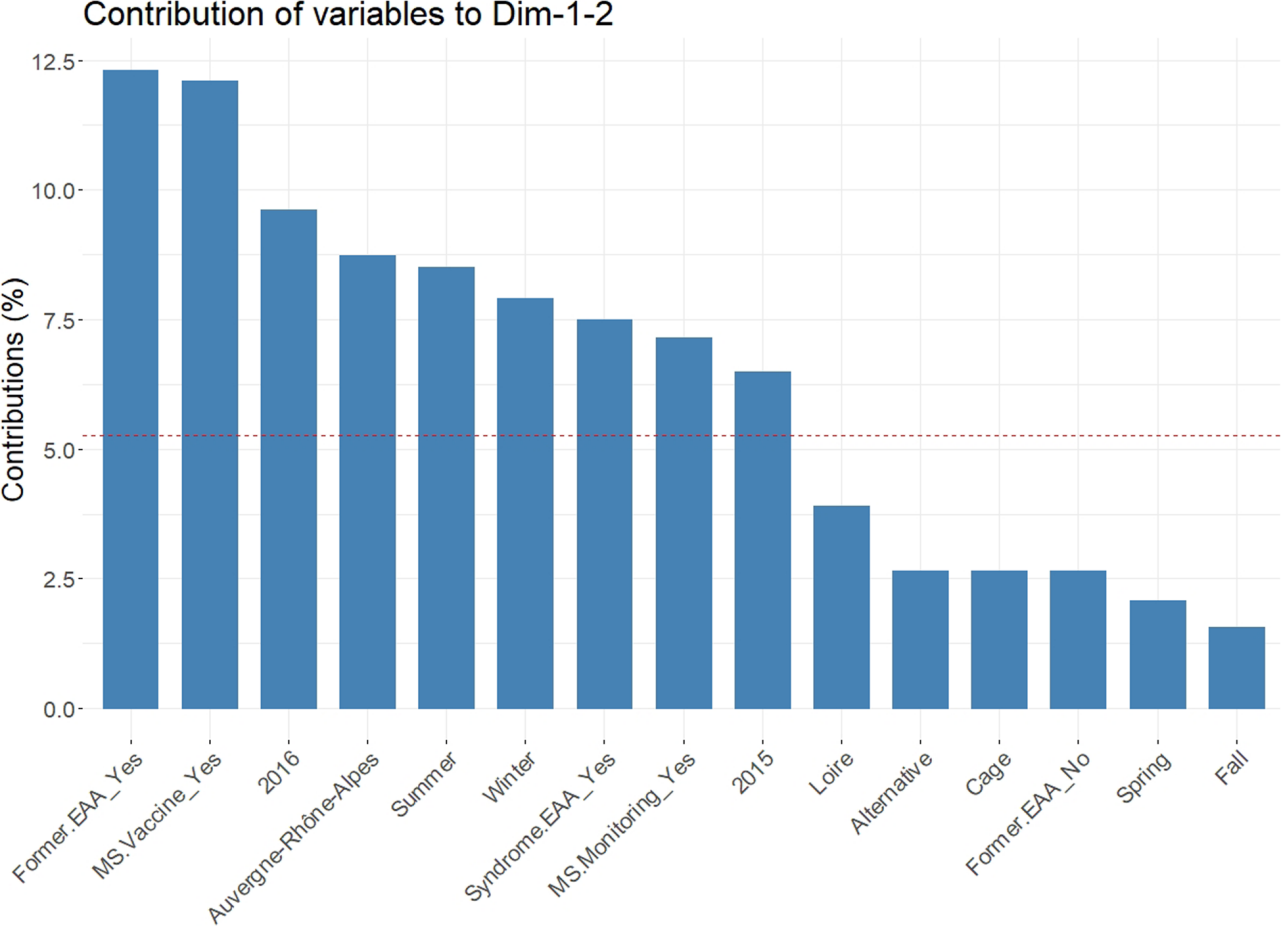
Graphical display of the contribution of the categories of variable for the first two dimensions of Multiple Correspondence Analysis (MCA). The dashed red line shows the expected mean value under the null hypothesis. Variables: Type of farm (Alternative or Cage), Area (Brittany, Loire, Auvergne-Rhône-Alpes), Season (Spring, Summer, Fall, Winter), Year (2015, 2016), Former.EAA (Yes, No), Syndrome.EAA (Yes, No), MS.Vaccine (Yes, No), MS.Monitoring (Yes, No)

Non dependence between variables studied in this survey on the past 5 years was demonstrated using a multiple correspondence analysis (MCA). For this analysis, in order to get more reliable and interpretable modalities of the date, real values of the date of survey were replaced with the season they belong to. The MCA results are illustrated on Fig. 1. The cumulated percentage of inertia explained with the first two dimensions is equal to 39.1%. The contributions of the categories of variables are given in Fig. 2. It follows that nine categories have a significant contribution to the two-dimensional representation: Former.EAA_Yes, MS.Vaccine_Yes, 2016, Auvergne-Rhône-Alpes, Summer, Winter, Syndrome.EAA_Yes, MS.Monitoring_Yes and 2015. More precisely, it is interesting to notice that “MS.Vaccine_Yes” is highly related (probability value (*p*-value) < 0.001) with “Former.EAA_Yes”: no EAA problems were detected in current flocks of farms that used MS vaccination after a former EAA-positive flock. One can also notice that “MS.Monitoring_Yes” is highly related (*p*-value< 0.05) with “Former.EAA_Yes” and “Syndrome.EAA_No”: farms detected EAA-positive in the last 5 years and that used laboratory tests for MS monitoring did not show clinical signs of EAA syndrome in the present flocks.

### Laboratory results (phase 2): MS detection using PCR

Different types of samples from 28 cases of EAA syndrome clinically detected by veterinarians were sent to our laboratory from January 2015 to June 2017: samples included fresh eggs, tracheal and cloacal swabs, originating from six regions of France (Table 2). *M. synoviae* was detected by PCR in 25/28 cases (89.3%) (Table 2), significantly more frequently from tracheal swabs (321/544 positive samples, 59.0%) than cloacal swabs (39/371 positive samples, 10.2%) (*p*-value < 0.001) (Table 3). For eggs, MS was more frequently detected from albumens (4/111 positive samples, 3.6%) than egg yolk (1/110 positive samples, 0.9%), but this difference was not statistically significant (*p*-value = 0.68). *M. synoviae* was detected in tracheal swabs of 23/24 (95.8%) EAA-positive cases and in cloacal swabs of 10/14 (71.4%) EAA-positive cases.

**Table 2:** Information on samples collected in layer flocks during phase 2 (2015–2017)

Case number	Region^**a**^	Production system^**b**^	Layer age (in weeks)	Sample type (swabs)	Number of samples collected	MS PCR detection^**c**^
1	IF	FC	52	trachea/cloaca	30/30	26/15
2	ARA	ALT	62	albumen/yolk	15/15	3/0
3	PACA	FC	60	trachea/cloaca	30/30	13/1
4	PACA	FC	54	albumen/yolk	30/30	0/0
5	B	FC	52	albumen/yolk	34/34	1/0
6	B	FC	56	trachea/cloaca	12/12	9/2
7	NA	FC	56	trachea/cloaca	32/32	24/1
8	B	FC	58	trachea/cloaca	30/30	25/2
9	B	FC	60	trachea/cloaca	30/30	20/1
10	B	FC	65	trachea/cloaca/albumen/yolk	30/30/9/9	9/1/0/0
11	ARA	ALT	12	trachea	10	9
12	BFC	FC	42	trachea	15	11
13	ARA	ALT	40	trachea/albumen	20/1	18/0
14	BFC	FC	51	trachea	20	17
15	ARA	FC	36	albumen/yolk	10/10	0/0
16	ARA	FC	64	trachea/cloaca	30/30	20/1
17	BFC	FC	62	trachea/cloaca	21/21	10/0
18	B	FC	60	trachea/cloaca	30/30	20/7
19	B	FC	68	trachea/cloaca	30/30	0/0
20	ARA	FC	60	trachea/cloaca/albumen/yolk	21/21/12/12	2/2/0/1
21	BFC	FC	49	trachea/cloaca	20/15	15/2
22	ARA	FC	52	trachea	15	12
23	B	ALT	61	trachea	15	4
24	B	ALT	60	trachea	9	1
25	ARA	FC	18	trachea/cloaca	30/30	17/4
26	B	FC	18	trachea	15	14
27	B	FC	24	trachea	18	11
28	B	FC	34	trachea	31	14

^a^*ARA* Auvergne-Rhône-Alpes, *B* Bretagne, *BFC* Bourgogne-Franche-Comté, *IF* Ile de France, *NA* Nouvelle Aquitaine, *PACA* Provence-Alpes-Côte d’Azur

^b^*FC* furnished cages, *ALT* alternative systems (barn, free-range, organic)

^c^ Number of MS positive samples (MS PCR performed on initial suspensions and after culture)

**Table 3:** *M. synoviae* detection by PCR in tracheal, cloacal, albumen and egg yolk swabs

Samples	ALT^**a**^		FC^**a**^		Total samples
	Farms	Samples	Farms	Samples	
Trachea	4/4^**b**^ (100%)	32/54^**c**^ (59.3%)	19/20^**b**^ (95.0%)	289/490^**c**^ (59.0%)	321/544^**c**,**d**^ (59.0%)
Cloaca	NS	NS	10/14 (71.4%)	39/371 (10.5%)	39/371^**e**^ (10.2%)
Albumen	1/1 (100%)	3/16 (18.8%)	1/5 (20.0%)	1/95 (1.1%)	4/111^**f**^ (3.6%)
Egg yolk	1/5 (0.0%)	0/15 (0.0%)	1/5 (20.0%)	1/95 (1.1%)	1/110^**f**^ (0.9%)
Total	5/5 (100%)	35/85 (41.2%)	20/20 (100%)	330/1051 (31.4%)	365/1136 (32.1%)

^a^*ALT* alternative systems (barn, free-range, organic), *FC* furnished cages

^b^ number of MS-positive farms/number of sampled farms (percentage of positive farms)

^c^ number of MS-positive samples/total number of samples (percentage of positive samples)

^d,e,f^ results with different letters are statistically different (*P* < 0.001)

*NS* not sampled

*M. synoviae* was detected in 5/5 ALT and 20/23 FC farms with EAA in the six different regions where flocks were sampled (Table 2). In ALT farms, MS was detected from 59.3% of tracheal and 18.8% of albumen swabs. In FC farms, MS was detected from 59% of tracheal and 10.5% of cloacal swabs, and 1.1% of albumen or egg yolk swabs (Table 3).

### *M. synoviae* isolation by in vitro cultivation

During this 30 months field study, clones were obtained from different cultures that were found MS-positive by PCR. However, isolation of MS clones was made difficult not only by the presence of bacterial contaminations but also by the presence of another *Mycoplasma* species in many samples, growing faster than MS in vitro. Assays of cultivation in presence of erythromycin neither prevented the growth of this *Mycoplasma* species nor allowed better isolation of MS isolates. Analyses performed by Matrix-Assisted Laser Desorption Ionisation – Time-of-Flight mass spectrometry (MALDI-TOF MSP) on 14 clones identified this other species as *Mycoplasma pullorum*. These results were confirmed based on sequence similarity (98.2 to 99.4%) of 16S rRNA gene of 14 clones with reference sequence of *M. pullorum* (U58504.1). Similarly, alignment of a subset of reads of these 14 clones against local nucleotide database gave a high percentage of similarity with the published complete sequence of *M. pullorum* (CP017813.1). These 14 Whole Genome Shotgun projects have been deposited at DDBJ/ENA/GenBank under the accession numbers PSYD00000000 to PSYQ00000000. The versions described in this paper are versions PSYD01000000 to PSYQ01000000.

Further analyses by MALDI-TOF MSP on cultures showed that *M. pullorum* was present in at least 14/25 flocks (56%) sampled during this study (Table 4). In total, 38/71 clones were identified as *M. pullorum* by MALDI-TOF MSP: four cloacal and 24 tracheal clones from FC flocks, one yolk sac and nine tracheal clones from ALT flocks. Additionally 33/71 clones were identified as MS by PCR: three cloacal and 30 tracheal clones from FC flocks.

**Table 4:** Isolation of *Mycoplasma* clones from tracheal, cloacal and egg yolk swabs

Samples	ALT^**a**^		FC^**a**^		
	MS^**b**^	MP^**b**^	MS^**b**^	MP^**b**^	Total clones
Trachea	0	9	30	24	63
Cloaca	0	0	3	4	7
Egg yolk	0	1	0	0	1
	0	10	33	28	71

^a^*ALT* alternative systems (barn, free-range, organic), *FC* furnished cages

^b^*MS M. synoviae*, *MP M. pullorum*

## Discussion

*M. synoviae* seroprevalence in layers is high worldwide [10, 11, 16, 23, 28, 31–35]. Surveys performed in France reported a seroprevalence of 60 and 68% for MS in laying hen flocks in 1999 and 2006, respectively [18, 36]. This high MS prevalence may lead to EAA clinical signs in layers [5]. Since the first observation of the EAA syndrome in 2008 in The Netherlands [5], EAA was detected in different countries including Japan, United Kingdom, Italy, Germany and Korea [5, 12, 13, 26, 37]. In France, the first case of EAA was described in 2009 [14]. However, the prevalence of this syndrome had never been precisely calculated. The present study demonstrated the prevalence of EAA syndrome in the French laying hen flocks (7.3% prevalence in the studied sample size). These results are in accordance with a previous study conducted with veterinarians, which reported 3 to 12% prevalence of EAA syndrome in France [38]. The field study also showed that the EAA syndrome is present in different production systems (furnished cages or alternative systems), with a higher presence in ALT farms. These results are in accordance with case reports describing EAA in FC or ALT systems [5, 12, 26]. The production conditions in ALT systems offer a lower bird density per square meter, a good ventilation and the access to open air via outdoor courses. On the other hand, access to an outdoor course increases the risk of exposure to MS infections, because biosecurity measures cannot be as strict as in closed buildings. Presence of MS in backyard chickens [29, 35, 39, 40], wild birds [8, 41, 42] or in other neighboring farms can therefore be a higher source of infection [10, 15, 25]. Multi-age farms can also increase the risk of infection between flocks in ALT or FC systems [9, 10, 18]. *M. synoviae* is indeed able to survive in the environment for several days, thus increasing the risk of contamination of flocks by indirect transmission [25, 43, 44].

This work also underlines the possible role of MS vaccination to decrease EAA clinical signs in layer flocks, which is in accordance with previous results showing that MS vaccination could decrease the incidence of EAA-associated lesions and improve flock’s performances [24, 25, 45, 46] then remove and reduce EAA clinical signs in layers [24]. However, it cannot be excluded that the significant difference evidenced in this survey between vaccinated and unvaccinated flocks may not be due in part to the introduction of more stringent biosecurity measures after the EAA problems encountered in the previous flock, or to other factors. *M. synoviae* vaccination was not a common practice at the time of the survey since only 25% of the EAA-positive farms visited in this study (5% of all the visited farms) used vaccination. This observation is compatible with that reported by Moreira and collaborators in Portugal [10]. Moreover, vaccination with autogenous killed vaccines or MS live vaccine was used only in farms with EAA problems in a former flock.

Laboratory monitoring was another variable studied in this field study and was shown to be statistically linked to the absence of EAA in current flocks. This result might be explained by the fact that farmers that have had a problem of MS infection with development of EAA in a former flock were more sensitive to the MS problem and performed tests to detect the problem earlier, with implementation of treatments (antimicrobial treatments, vaccination, better disinfection between flocks) or stricter biosecurity measures than farmers that did not experience EAA problems before.

One of the limitations of the current study is that the results were based on the farmers’ responses instead of laboratory tests. It is possible that there could be other factors than MS infection alone that might be affecting egg quality, such as infectious bronchitis as suggested by Gole et al. [16]. However, the second part of the study showed that in most clinical cases of EAA, laboratory analyses demonstrated that MS was present in the flocks.

For the laboratory study, detection of MS DNA and isolation of MS clones were conducted over a 3 year period, from 2015 to 2017: sampling was performed by veterinarians in 28 EAA-positive farms of six regions of France. Although the field survey focused on layers over 59 weeks of age, samples for the laboratory study were collected in flocks at different stages of production: 25 layer flocks in production (22 to 68 weeks of age) and three flocks of future layers in multi-age farms with recurrent problems of EAA (12 to 18 weeks of age).

Results showed that tracheal swabs were the most frequent MS-positive samples by PCR and culture in EAA-positive flocks compared to cloacal or egg swabs. This high level of MS detection in tracheal swabs is in accordance with previous studies [10, 12, 18, 47]. The difference between tracheal and cloacal swabs may be explained by the fact that MS is known to have a strong tropism for tissues of the respiratory tract [8] and that its cloacal excretion may be too low (under the detection threshold), intermittent or too irregular, as already suggested by Marois et al. [22]. Contaminations by other bacteria and PCR inhibitors may contribute to lower detection results in cloacal samples [22]. However, Ranck et al. [37] showed that cloacal swabs from hens of EAA-positive flocks were more frequently detected positive than cloacal swabs from hens of EAA-negative flocks. *M. synoviae* recovery or detection might have been improved by taking samples directly from the reproductive tract of laying hens and not from cloaca, but our study was based on routine collection of samples from live animals following field veterinary practices for final diagnosis. Studies performed on oviductal swabs of hens from EAA-positive flocks at necropsy showed a good MS detection level by PCR (33 to 80%) but isolation of MS strains was more difficult than from tracheal samples [12, 48]. It should be noted however that isolation of MS from tracheal swabs is not directly related to the development of the syndrome: only a few MS-infected flocks are affected by EAA and appearance of abnormal eggs is associated with the presence of MS at the oviduct level [5, 49].

Results of our study also showed that MS could be detected in most of the farms with EAA clinical signs (25/28 sampled flocks, 89.3%). For two of the three FC farms where MS was not detected, eggs were the only samples sent for laboratory analyses. Since MS detection and isolation from egg samples (albumens or yolk sacs) was significantly lower than from cloacal and tracheal swabs, it might explain the non detection of MS in these flocks. This low percentage of infected eggs found in our study is in accordance with studies on MS vertical transmission showing that only 3 to 10% of eggs are infected by MS after a natural or experimental infection of hens [50–53]. However, Catania et al. [12] and Ranck et al. [37] were able to detect MS DNA in 53.3 and 98% of fresh eggs from EAA-positive flocks, respectively.

In this study, the onset of EAA clinical signs was observed in all stages of production and suggested that the egg lesions observed did not depend on the age of the hen. Very little information about age of EAA onset is available in previous studies: Strugnell and collaborators reported that incidence of abnormal eggs began to increase at around week 42 [26] and Jeon and collaborators reported an EAA outbreak in 54 week-old layers [13]. The hen’s age could also affect the egg production and quality [54]; however, in our study, samples were collected from clinical cases representing various age groups.

Isolation of MS clones from cultures of tracheal, cloacal or egg swabs was hampered by the presence of another *Mycoplasma* species in many samples, growing faster than MS in vitro. Assays performed with FM4 broth medium supplemented with erythromycin failed to arrest the growth of this *Mycoplasma* species, suggesting its resistance to erythromycin. Analyses performed by MALDI-TOF MSP on 14 clones identified this species as *M. pullorum*. This identification was confirmed by analysis of whole genome sequences obtained on these 14 clones.

Results of in vitro culture with erythromycin are in accordance with results of Whithear et al. [55] showing that 5/5 strains of *M. pullorum* tested were resistant to erythromycin (MIC of 80 μg/mL), but susceptible to tylosin, suggesting a natural resistance like MS to 14-membered ring macrolides [56]. The classification of *M. pullorum* in the Hominis group is another factor that leans toward innate resistance of this species to 14-membered ring macrolides like other *Mycoplasma* species of this group (*M. synoviae*, *M. hyopneumoniae*, *M. hyorhinis* and *M. bovis* for example) [56, 57].

The MALDI-TOF MSP is increasingly used for bacterial identification, but very few studies have been published on its adaptation to different *Mycoplasma* species [58–61]. In our study, the identification of 14 *M. pullorum* strains by both NGS and MALDI-TOF MSP allowed us to validate the MALDI-TOF MSP technique for routine use within our laboratory on cultures and clones for identification of this *Mycoplasma* species, because no *M. pullorum*-specific PCR test has been published yet for its rapid detection. This method allowed cheaper, easier and faster identification of *M. pullorum* than biochemical methods or sequencing and should be considered as a reliable method for identification of this species. Further analyses by MALDI-TOF MSP on cultures and clones showed that *M. pullorum* was present in 56% of samples collected for this study.

Very few studies have been published on *M. pullorum*. It was isolated from pheasants and partridges with signs of upper respiratory disease but was considered as a fast-growing saprophytic *Mycoplasma* species impeding the isolation of another pathogenic *Mycoplasma* [41]. However, *M. pullorum* has been isolated alone or with other *Mycoplasma* species from trachea or air-sac lesions of chickens with respiratory problems [62, 63] and from dead chicken embryos [62]. *M. pullorum* was also identified in dead turkey embryos [64]. Isolation of clones from egg and cloacal swabs in our study and from chicken and turkey embryos in previous published studies strongly suggests the possibility of genital tropism and therefore vertical transmission of *M. pullorum*. Further studies (experimental infections and/or field surveys, development of a specific PCR to detect this species in samples without a cultivation step) need to be conducted to test whether *M. pullorum* might play a role, as already demonstrated for Infectious Bronchitis virus [5], in the exacerbation of clinical signs of EAA in MS-infected layer farms.

## Conclusion

In conclusion, laboratory results confirmed data showed by the field survey: the EAA syndrome induced by MS is present among different layer production systems (furnished cages and alternative systems) and regions of France with a prevalence of 7.3% in the studied sample. Subsequent studies are necessary to determine if *M. pullorum* could play a role in the expression of the clinical signs of the EAA syndrome in case of co-infection with *M. synoviae*.

## Methods

### Field survey (phase 1)

A 12-month field survey was performed between May 2015 and May 2016. Sample size for this survey was calculated considering a population of 46 millions of commercial layers in 2100 farms, with 70% of laying hens housed in furnished cages (FC) and 30% in alternative systems (ALT). Three geographical zones concentrating 62% of commercial layer farms in France were selected: Bretagne, Pays de la Loire and Auvergne-Rhône-Alpes. The two systems of production (ALT and FC) were considered for this study as pathogen contamination pressures may be different between these two systems. This segmentation allowed improvement of the accuracy of the results. The study unit was the flock: a layer population with the same origin and placed in the same house at the same time. The number of flocks to be studied was calculated based on the total number of French laying hen flocks (*n* = 2100) and on an expected prevalence of 9% [38], at +/− 5% and with a risk alpha of 5% [65], thus a total of 126 farms were included in the survey. The survey presentation to farmers had an introduction about the main clinical signs of EAA syndrome (increased incidence of soft-shelled eggs and egg breakage on harvesting mats and other facilities, with abnormalities confined to the top cone of the egg, up to approximately 2 cm from the apex, and almost always with a very clear demarcation zone), with pictures of eggs with typical lesions of EAA (cap about 2 cm around the apex, thinning of the shell at the apex level with cracks and breaks) for better identification of positive cases in current or past flocks. The questionnaires were recorded individually during the investigator’s visit with farmers who had layers of more than 59 weeks of age, to ensure that the information collected was after a nearly complete production cycle. The independent variable was the presence or absence of EAA in flocks under study. A flock was classified as EAA-positive if clinical signs characteristic of this syndrome (apex with altered and thinner shell surface and increased translucency) were observed. The dependent variables were: (i) the flock’s age at the beginning of EAA syndrome, (ii) the application or not of any vaccine against MS (autogenous or commercial) and (iii) MS monitoring or not throughout the production life of the flock.

### Field sample collection for laboratory analysis (phase 2)

To detect the causative agent of the EAA syndrome, 1136 samples (544 tracheal, 371 cloacal, 111 albumen, and 110 egg yolk swabs) were collected by veterinarians from 28 flocks of different ages in six regions of France (Table 2): 25 layer flocks in production (between 22 and 68 weeks of age) with clinical signs of EAA and three flocks of future layers in farms with recurrent problems of EAA (between 12 and 18 weeks of age) were sampled. These samples were delivered to our laboratory for diagnosis from January 2015 to June 2017. Ethical approval was not required for the study because samples were collected during routine diagnostic examinations by veterinarians with the consent of the farmers (Article 1, 5a of Directive 2010/63/EU).

### Isolation of MS by in vitro cultivation

All swabs were placed in 2 mL of Frey Medium 4 (FM4) broth [66] supplemented with antimicrobials (Amphotericin B (Sigma-Aldrich) 25 μg/mL, Ampicillin (Sigma-Aldrich) 2 units/mL and Colistin (Sigma-Aldrich) 75 mg/mL) to obtain initial suspensions. Mycoplasmas were directly cultured by diluting 100 μL of initial suspension from each sample in 900 μL of FM4 broth supplemented with antimicrobials and serial dilutions up to 10^− 3^ were performed. All dilutions were incubated at 37 +/− 2 °C until the culture developed an acid color change or for a maximum of 30 days. Since MS is naturally resistant to erythromycin [56], cultures were also performed in FM4 broth medium supplemented with different erythromycin (Sigma-Aldrich) concentrations ranging from 4 to 10 μg/mL when isolation of MS was made difficult due to the presence of other *Mycoplasma* species. Subcultures onto FM4 agar were performed after the development of an acid color change and agar plates were incubated at 37 +/− 2 °C with 5% CO_2_ for 5–10 days. Clones were obtained by picking single *Mycoplasma* colonies under a stereomicroscope and growing them in FM4 broth as described above. Initial suspensions, cultures, and clones were stored at ≤ − 65 °C in 20% glycerol until further analysis.

### *M. synoviae* DNA detection using polymerase chain reaction (PCR)

Deoxyribonucleic acid (DNA) samples were prepared by cellular lysis according to the method of Kellog and Kwok [67]. Briefly, 1 mL of initial suspensions, cultures or clones were centrifuged at 12,000 x *g* for 15 min and the pellets were suspended in a mixture of 250 μL of solution A (100 mM KCl, 10 mM Tris-HCl (pH 8.3), 2.5 mM MgC1_2_) and 250 μL of solution B (10 mM Tris-HCL (pH 8.3), 2.5 mM MgCl_2_, 1% Tween 20 and 1% Nonidet P40). Samples were incubated for 60 min at 60 °C prior to proteinase K heat-inactivation at 95 °C for 15 min, allowed to cool at room temperature and kept at 5 +/− 3 °C. The presence of MS DNA in samples and cultures was detected by a MS-specific PCR as previously described by Lauerman et al. [68] in a final volume of 50 μL. Briefly, the PCR mixture contained 200 mM of each primer (MSL1 5′-GAGAAGCAAAATAGTGATATCA-3′ and MSL2 5′-CAGTCGTCTCCGAAGTTAACAA-3′), 200 μM of each deoxyribonucleotide triphosphate (Eurobio), 5 μL of 10X PCR buffer (Roche), 2 mM of MgCl_2_ (Bio-Rad), 1.25 unit of Taq polymerase (Roche) and 5 μL of DNA samples. A negative control (water, molecular biology grade, Eurobio) and a positive control (MS reference strain WVU 1853) were added to each PCR assay. PCR were performed with a thermal cycler (T100™ Bio-Rad): 94 °C for 5 min, 35 cycles at 94 °C for 1 min, 50 °C for 1 min, and 72 °C for 2 min, followed by an elongation step at 72 °C for 5 min. Amplified DNA products were separated in a 2% agarose gel in Tris-Borate-ethylenediaminetetraacetic acid (EDTA) (TBE) buffer (90 mM Tris, 90 mM borate, 2.5 mM EDTA pH 8.0) for 1 h at a constant voltage of 110 V. Amplified products were detected by ultraviolet transillumination with ethidium bromide staining. A GeneRuler 1 kb DNA Ladder (Thermo Fisher Scientific) was used as a molecular size standard: the expected size of MS amplicon was 207 bp.

### MALDI-TOF mass spectrometry (MALDI-TOF MSP) analysis

Clones were thawed, diluted 1/10 and grown in FM4 broth medium in a final volume of 3 mL. *Mycoplasma* pellets, obtained after centrifugation at 20,000 x *g* for 15 min, were washed twice in 1X phosphate-buffered saline and suspended in 300 μL of water and 900 μL of absolute ethanol to precipitate proteins. After centrifugation at 20,000 x *g* for 2 min, the supernatants were removed and the pellets were dissolved in 30 μL of 70% formic acid (Sigma-Aldrich) and 30 μL of acetonitrile (Sigma-Aldrich). After centrifugation for 2 min at 20,000 x *g*, 1 μL of supernatant was spotted onto a MALDI-TOF MSP 96 target polished steel plate (Bruker Daltonics). After air-drying at room temperature, each sample was overlaid with 1 μL of matrix solution: α-cyano-4-hydroxycinnamic acid (HCCA, Bruker Daltonics) solubilized in standard solvent (50% acetonitrile, 47.5% water and 2.5% trifluoroacetic acid, Sigma-Aldrich) to obtain a 10 mg/mL solution. Samples were air-dried at room temperature before MALDI-TOF MSP analysis. Spectra were generated using a Microflex LT Biotyper operating system (Bruker Daltonics). The data was analyzed by the Bruker Biotyper 3.0 software and the Bruker taxonomy library. The degree of spectral concordance was expressed as a logarithmic identification score ranging from 0 to 3 and was interpreted according to the manufacturer’s instructions, with a modification of the score that was acceptable for probable species-level identification, which was lowered from ≥2.000 to ≥1.700 [60].

### Next-generation sequencing (NGS) of clones

Several clones giving a *M. pullorum* identification result with MALDI-TOF MSP were sequenced for *Mycoplasma* species confirmation. DNA was extracted from a 12 mL culture with the QIAamp® DNA Mini Kit (Qiagen) and quantified with a Qubit® 2.0 fluorometer (Invitrogen). DNA was sheared by sonication using a Bioruptor® Plus (Diagenode) apparatus. Libraries were prepared using NEBNext Ultra DNA library Prep Kit for Illumina and NEBNext Multiplex Oligos for Illumina according to the manufacturer’s instructions (New England Biolabs). Size selection and purification steps were conducted with magnetic beads Agencourt AMP pure XP (Beckman-Coulter). Sequencing was performed using Mi-seq Illumina technology (paired-end sequencing 2 × 150 cycles, MiSeq Reagent kit v2–300 Cycles, Illumina). After cleaning with Trimmomatic 0.36 [69] (ILLUMINACLIP:oligos.fasta:2:30:5:1:true LEADING:28 TRAILING:28 MAXINFO:40:0.2 MINLEN:36), the 16S rRNA gene and a subset of reads were aligned against local nucleotide database (Megablast 2.2.26).

### Statistical analysis

A Multiple Correspondence Analysis (MCA) [70] was applied to the whole data set to illustrate the underlying relationships between the eight categorical variables under study in a low-dimensional Euclidean space. In addition, the observations on which the variables were measured were also plotted and interpreted simultaneously. Observations and categories of the variables were plotted on the same graphical display while using the quasi-barycentric property. The optimal dimension of the Euclidean space to be interpreted was derived from the associated eigenvalues (i.e., transformed into proportion of inertia). Associations between the categories of the variables were uncovered by calculating the chi-square distances [71]. To get more interpretable modalities of the date (i.e., limited number of categories), real values of the date of survey were replaced with the season they belong to. The statistical analysis was performed with the R software [72] version 3.6.1 using the “FactoMineR” [73] and “factoextra” packages [74]. A significance level (*p*-value) of 5% was used.

## Data Availability

The 14 Whole Genome Shotgun projects generated and analysed during the current study have been deposited at DDBJ/ENA/GenBank under the accession numbers PSYD00000000 to PSYQ00000000 (ncbi.nlm.nih.gov). Other datasets used and/or analysed during the current study are available from the corresponding author on reasonable request.

## Abbreviations

ALT: Alternative systems
Bp: Base pair
CI: Confidence interval
°C: Degree Celsius
DDBJ/ENA/GenBank: DNA Databank of Japan / European Nucleotide Archive / GenBank
DNA: Deoxyribonucleic acid
EAA: Eggshell apex abnormality
EDTA: Ethylenediaminetetraacetic acid
ELISA: Enzyme-linked immunosorbent assay
FC: Furnished cages
FM4: Frey medium 4
*g*: Gravitational acceleration
h: Hour
HCCA: α-cyano-4-hydroxycinnamic acid
kb: Kilobase
*M.*: *Mycoplasma*
MALDI-TOF MSP: Matrix-assisted laser desorption ionisation – time of flight mass spectrometry
MCA: Multiple correspondence analysis
min: Minute
μL: Microlitre
mL: Millilitre
mM: Millimolar
MS: *Mycoplasma synoviae*
NGS: Next-generation sequencing
OIE: World Organisation for Animal Health
PCR: Polymerase chain reaction
*p*-value: Probability value
RSA: Rapid serum agglutination
TBE: Tris, Borate, EDTA
V: Volt
